# Anti-inflammatory effect of herbal traditional medicine extract on molecular regulation in allergic asthma 

**DOI:** 10.5414/ALS400545

**Published:** 2021-04-16

**Authors:** Xiaopeng Sun, Entezar Mehrabi Nasab, Seyyede Masoume Athari, Seyyed Shamsadin Athari

**Affiliations:** 1 Department of Otolaryngology, Second Affiliated Hospital of Xi’an Medical College, Xi’an, China,; 2 Cardiologist, Department of Cardiology, School of Medicine, Tehran Heart Center, Tehran University of Medical Sciences, Tehran,; 3 Department of Biology, Faculty of Basic Sciences, Maragheh University, Maragheh, and; 4 Department of Immunology, School of Medicine, Zanjan University of Medical Sciences, Zanjan, Iran

**Keywords:** allergy, asthma, herb, pathophysiology, immune response

## Abstract

Asthma is an important global health problem, and the main cause of asthma is allergic reaction and immune system dysregulation. Airway inflammation causes bronchial narrowing, and goblet cell hyperplasia leads to mucus hypersecretion that leads to airflow obstruction and difficulty breathing. The Th2 cytokines can induce allergic asthma. Camellia, Adhatoda, and Glycyrrhiza are the traditional medicines that are used in some countries. In the current study, we evaluated three herbal extracts on airway inflammatory responses in asthmatic mice. The asthma model was induced in mice that were divided into 6 groups: Phosphate-buffered saline (PBS) group, ovalbumin (OVA) group, OVA-budesonide group, OVA-Glycyrrhiza group, OVA-Camellia group, and OVA-Adhatoda group. Measurements of IL-4, IL-5, IL-13, glutamate oxaloacetate transaminase (GOT), glutamic pyruvic transaminase (GPT), IgE, histamine, percentages of eosinophils in bronchoalveolar lavage fluid (BALf), gene expression of COX-2, CCL24, CCL11, eotaxin, and histopathological study of lung were done. Adhatoda significantly attenuated the IL-4, IgE, and histamine levels. Glycyrrhiza attenuated the levels of IL-5, IL-13, GTP, GOT (on day 51), mRNA expression of eotaxin, CCL24, CCL11, and COX-2, eosinophil infiltration, mucus secretion, and goblet cell hyperplasia. Camellia decreased IL-13, GTP, COX-2 mRNA expression, mucus secretion, and goblet cell hyperplasia on day 31 and 51. We evaluated effect of three plants on allergic bio-factors. Glycyrrhiza as main anti-inflammatory treatment, Adhatoda as anti-allergic, and Camellia as anti-mucus releasing treatment can be used in attacks of allergic asthma.

## Introduction 

Asthma is an important global disease, with continually increasing prevalence and mortality. The main cause of asthma is allergic reaction and dysregulation of the immune response. Allergic asthma is a bronchial inflammatory disease characterized by wheezing, cough, and breathlessness. In asthmatic patients, bronchial inflammation causes airway narrowing, and goblet cell hyperplasia leads to mucus hypersecretion that blocks the airways. At the time of asthma attack, airway hyper-responsiveness (AHR) causes airflow obstruction, difficulty breathing, and may lead to death [1, 2]. 

Immune imbalance between Th1 and Th2 cytokines leads to over-activation of Th2 cytokine secretion (mainly IL-4, IL-5, and IL-13). These Th2-associated cytokines can induce allergic asthma. IL-4 forces B cells to produce IgE, and after sensitization and activation of mast cells and basophils, causes initiation of allergic reaction and release of allergic mediators. IL-5 can activate eosinophils and induce their migration and infiltration, inducing eosinophilic airway inflammation. IL-13 stimulates goblet cell hyperplasia and mucus hypersecretion, and can exacerbate airway obstruction [3, 4]. Infiltration and activation of inflammatory immune cells, especially eosinophils, increase the reactive oxygen species (ROS) levels and worsen inflammation and bronchial injury in lung tissue. These cells also release eosinophil peroxidase and eosinophil cationic protein, which cause the inflammatory reaction. Thus, regulation of the Th2-cell activity and decreased inflammation in asthma progression is important [5, 6]. 

Licorice or Glycyrrhiza is one of the traditional medicines used in some countries of Asia. It has multiple biological functions and can reduce the inflammatory response in tissues [7, 8]. Glycyrrhiza is one of the constituents in anti-asthma herbal medicine that has therapeutic effects in respiratory medicine. Also, Glycyrrhiza has eotaxin-1 inhibitory flavonoids [9], which can control eosinophilic inflammation. Glycyrrhizin can inhibit immediate bronchial constriction, AHR, and infiltration of eosinophils in the lung [10, 11]. *Camellia sinensis L.* (green tea) is a native plant of Asia that today is cultivated across the world. The leaves are used as a drinking supplement. The antioxidant and anti-inflammatory properties of *Camellia sinensis* have been studied [12]. Therefore, it may have anti-asthma effects, which is what we evaluated in this study. In China, *Adhatoda vasica* is a popular folk medicine that is used for the treatment of asthma and colds [13]. In the current study, we evaluated three herbal extracts with regard to the molecular mechanisms of airway inflammatory responses in asthmatic mice. 

## Materials and methods 

### Animals and treatment schedule 

Female 7-week-old BALB/c mice were purchased and raised in animal housing under standard conditions. Animal experiments were done according to the Laboratory Animal Care ethical guidelines. 72 mice were divided into 6 experimental groups to produce an allergic asthma model: negative control mice (healthy and normal group) that were sensitized and challenged with normal saline; the remaining 5 groups were sensitized and challenged with ovalbumin (OVA): OVA-sensitized control mice (asthma and positive control group); asthma mice treated with budesonide (0.5 mg/30 min inhalation); asthma mice treated with Glycyrrhiza extract (1 mg/1 mL/30 min inhalation); asthma mice treated with Camellia extract (1 mg/1 mL/30 min inhalation); asthma mice treated with Adhatoda extract (1 mg/1 mL/30 min inhalation). 

### Sensitization and administration 

**Figure 1.**
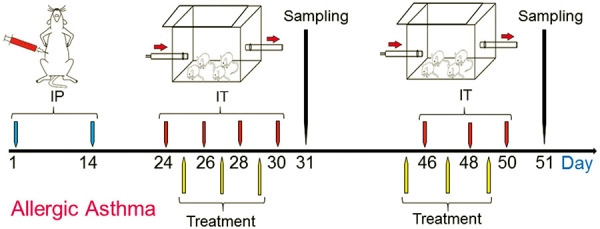
Allergic Asthma Animal Model. Mice were sensitized by intraperitoneal injection (IP) of ovalbumin (OVA) with alum on days 1 and 14, then challenged by IT of OVA solution on days 24, 26, 28, and 30 to produce a mouse model of asthma To maintain allergic asthma condition, IT of OVA solution were continued on days 46, 48, and 50. Treatment was done on day 25, 27, 29, 45, 47, and 49; sampling was done on days 31 and 51.

Mice sensitization and challenging is shown in Figure 1 as described previously [1, 14]. Briefly, mice were sensitized with intraperitoneal (IP) injections of OVA solution with alum on days 1 and 14, and challenged with inhalation administration (IT) of OVA solution by nebulizer for 30 minutes on days 24, 26, 28, 30, 46, 48, and 50. The mice were treated with three extracts in inhalation form for 30 minutes on days 25, 27, 29, 45, 47, and 49. Half of the mice in each group were euthanized on day 31 and the other half on day 51, and sampling (from blood, bronchoalveolar lavage fluid (BALf), and lung tissue) was done. 

### BALf cell counts 

BALf was collected from the trachea of mice via intubation and stored. Then BALf was centrifuged, and supernatant collected to analyze cytokine and chemokine levels. The cells of the BALf were used to produce slides with ccytocentrifugation, and were stained with Giemsa stain to determine the percentages of eosinophils. 

### Cytokines 

The levels of IL-4, IL-5, and IL-13 were measured by specific ELISA kits according to the manufacturer’s instructions (R&D, Newark, NJ, USA). 

### Serum biochemical analysis 

The glutamate oxaloacetate transaminase (GOT) and glutamic pyruvic transaminase (GPT) levels were detected in serum using a biochemical analyzer (FACA-301, Thomas Scientific, Newark, NJ, USA). 

### Serum immunoglobulin 

Blood samples were centrifuged to separate serum, and then total IgE level was measured by ELISA (BD Biosciences, Los Angeles, CA, USA) method. 

### Histamine level 

The histamine level was determined in serum by ELISA Kits (Biocompare, Newark, NJ, USA). 

### Quantitative real-time PCR 

RNA was extracted from BALf cells using TRI reagent, and cDNA was synthesized using a cDNA synthesis kit. Study expressions of the target genes were done using SYBR Green Master Mix (Bio-Rad, Los Angeles, CA, USA). The used primers are given in Table 1. 

### Histological study of the lung 

Lung tissues were fixed with formalin and then cut in slide sections for hematoxylin and eosin (H & E) and Alcian Blue-Periodic Acid Schiff (AB-PAS) staining. Then, under light microscopy, eosinophil infiltration around bronchi and vessels, goblet cell hyperplasia, and mucus secretion were evaluated and scored [11, 14]. 

### Statistical analysis 

Statistical analyses were performed using version 20 SPSS and ANOVA and a Dunnett post-hoc test. Our data were expressed as the mean ± SD of at least 3 independent experiments, and p < 0.05 was considered significant. Graphs were generated using GraphPad prism. 

## Results 

### Eosinophils in BALf 

**Figure 2.**
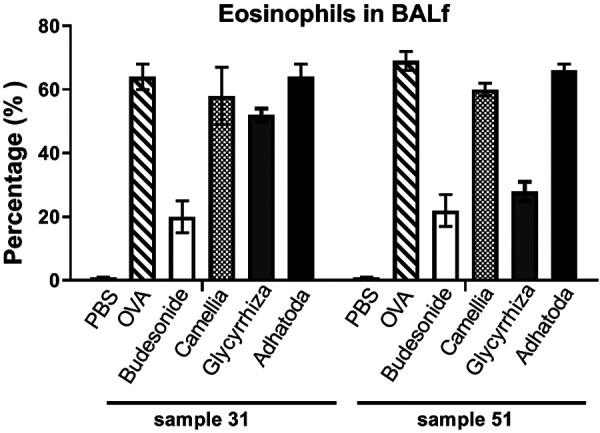
Eosinophil percentage in bronchoalveolar lavage fluid (BALf). The percentage of eosinophil was assessed in six groups on days 31 and 51 by cytocentrifugation method.

The percentage of eosinophils in the BALf were counted after cytocentrifugation. The OVA group had increased numbers of eosinophils in the BALf compared to the PBS group on days 31 (64 ± 4 vs. 4 ± 0.1%, p < 0.05) and 51 (69 ± 3 vs. 5 ± 0.1%, p < 0.05). The eosinophil counts were not significantly decreased by the three herbal treatments (Camellia: 58 ± 9, Glycyrrhiza: 52 ± 2, Adhatoda: 64 ± 4) on day 31, but they were significantly decreased by Glycyrrhiza treatment (28 ± 3) on day 51 (Figure 2). 

### Cytokines 

**Figure 3.**
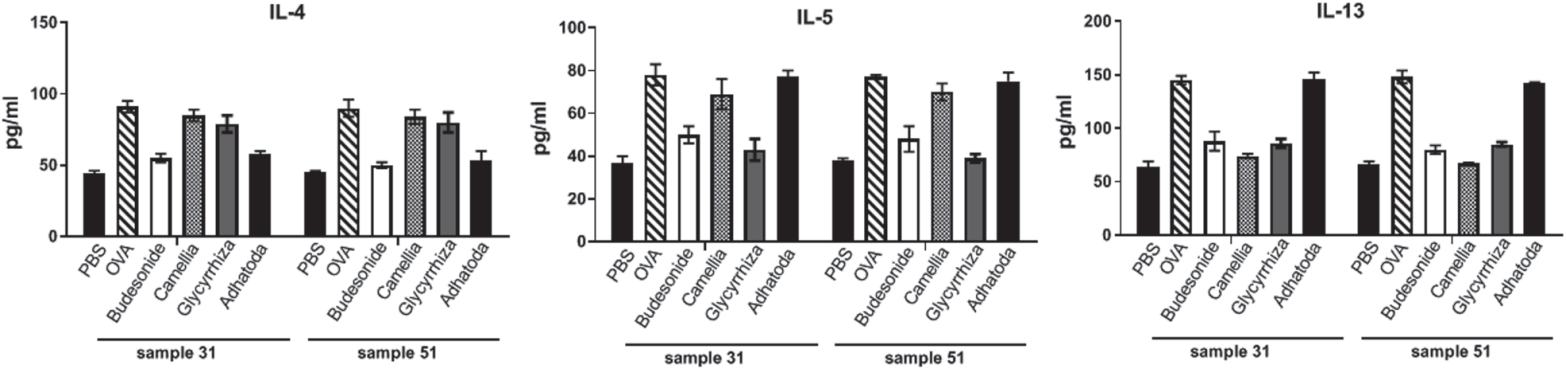
Cytokine levels. The levels of IL-4, IL-5, and IL-13 in bronchoalveolar lavage fluid were measured by ELISA in all 6 groups on days 31 and 51.

The levels of IL-4 (91 ± 4 vs. 90 ± 6 pg/mL, p < 0.05), IL-5 (78 ± 5 vs. 77 ± 1 pg/mL, p < 0.05), and IL-13 (145 ± 4 vs. 148 ± 6 pg/mL, p < 0.05) were increased in the asthma animals on days 31 and 51 compared with those seen in the healthy animals: IL-4 (44 ± 2 vs. 45 ± 1 pg/mL, p < 0.05), IL-5 (37 ± 3 vs. 38 ± 1 pg/mL, p < 0.05), and IL-13 (64 ± 5 vs. 66 ± 3 pg/mL, p < 0.05) (Figure 3). 

Treatment with Camellia (85 ± 4 vs. 84 ± 5 pg/mL, p < 0.05) and Glycyrrhiza (79 ± 6 vs. 80 ± 7 pg/mL, p < 0.05) did not affect OVA-induced IL-4 level on day 31 and 51, respectively. In contrast, Adhatoda significantly attenuated the level of IL-4 (day 31: 58 ± 2, day 51: 53 ± 7, pg/mL). Also, Glycyrrhiza significantly attenuated the levels of IL-5 (43 ± 5, 39 ± 2, respectively) and IL-13 (86 ± 4, 85 ± 2, respectively) on day 31 and 51, and Camellia significantly decreased IL-13 level on day 31 and 51 (74 ± 2, 67 ± 1, respectively) (Figure 3). 

### Serum biochemical analysis 

**Figure 4.**
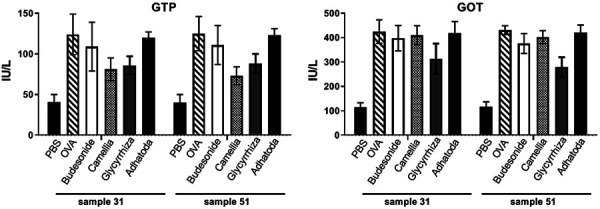
GTP and GOT levels. The levels of GTP and GOT were measured on days 31 and 51 in the 6 mentioned groups.

In serum, Glycyrrhiza and Camellia reduced the levels of GTP on days 31 and 51, but these reductions were not significant. Glycyrrhiza reduced GOT levels in asthmatic mice on day 51 significantly (Figure 4). 

### Serum immunoglobulin 

**Figure 5.**
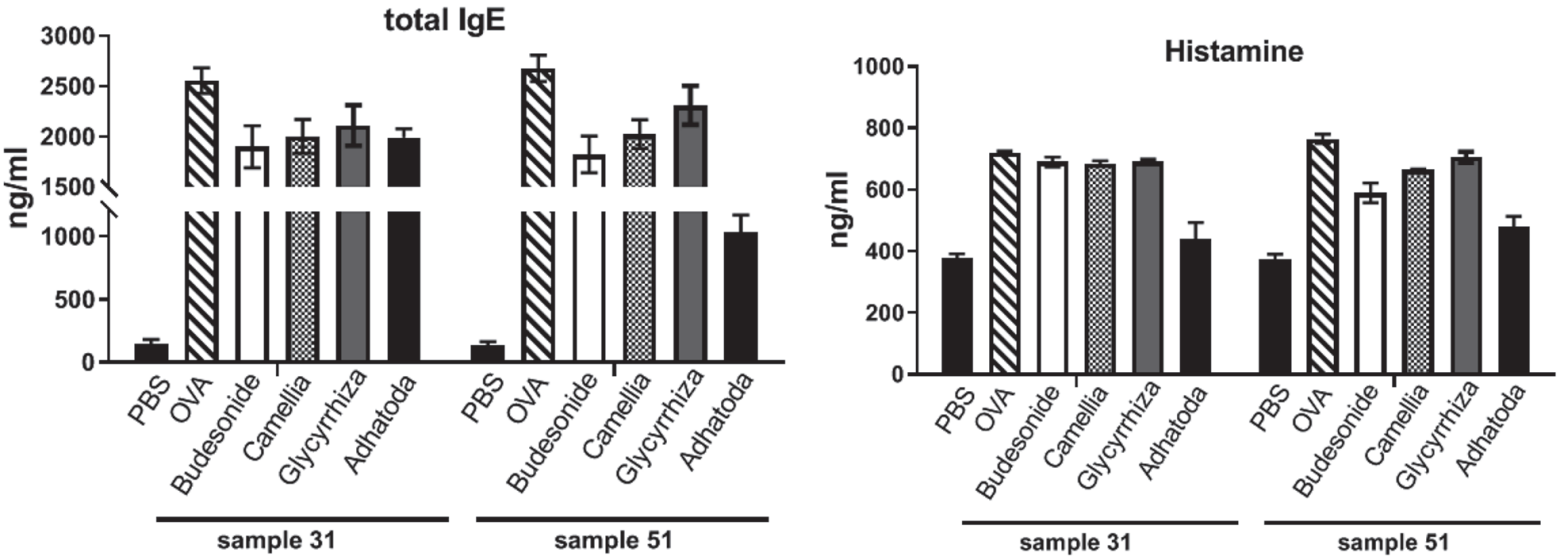
IgE and histamine levels. The levels of total IgE and histamine were measured on days 31 and 51 in the serum of the 6 groups.

In the serum of the OVA group, total IgE was significantly increased (day 31: 2,556 ± 127, day 51: 2,678 ± 132) compared to the PBS group (day 31: 146 ± 34, day 51: 141 ± 23) (p < 0.05). Glycyrrhiza and Camellia treatment had no significant effect on total IgE compared with OVA group on days 31 and 51. However, Adhatoda reduced the total IgE level in serum significantly (1,032 ± 139) on day 51 (p < 0.05) compared to the OVA group (Figure 5). 

### Histamine level 

In the OVA group, the histamine level was significantly increased compared to the PBS group on days 31 and 51 *(*p < 0.05). Glycyrrhiza and Camellia treatment had no significant effect on histamine level compared with the OVA group. However, Adhatoda decreased histamine level significantly on day 31 and 51 *(*p < 0.05) (Figure 5). 

### Quantitative real-time PCR 

**Figure 6.**
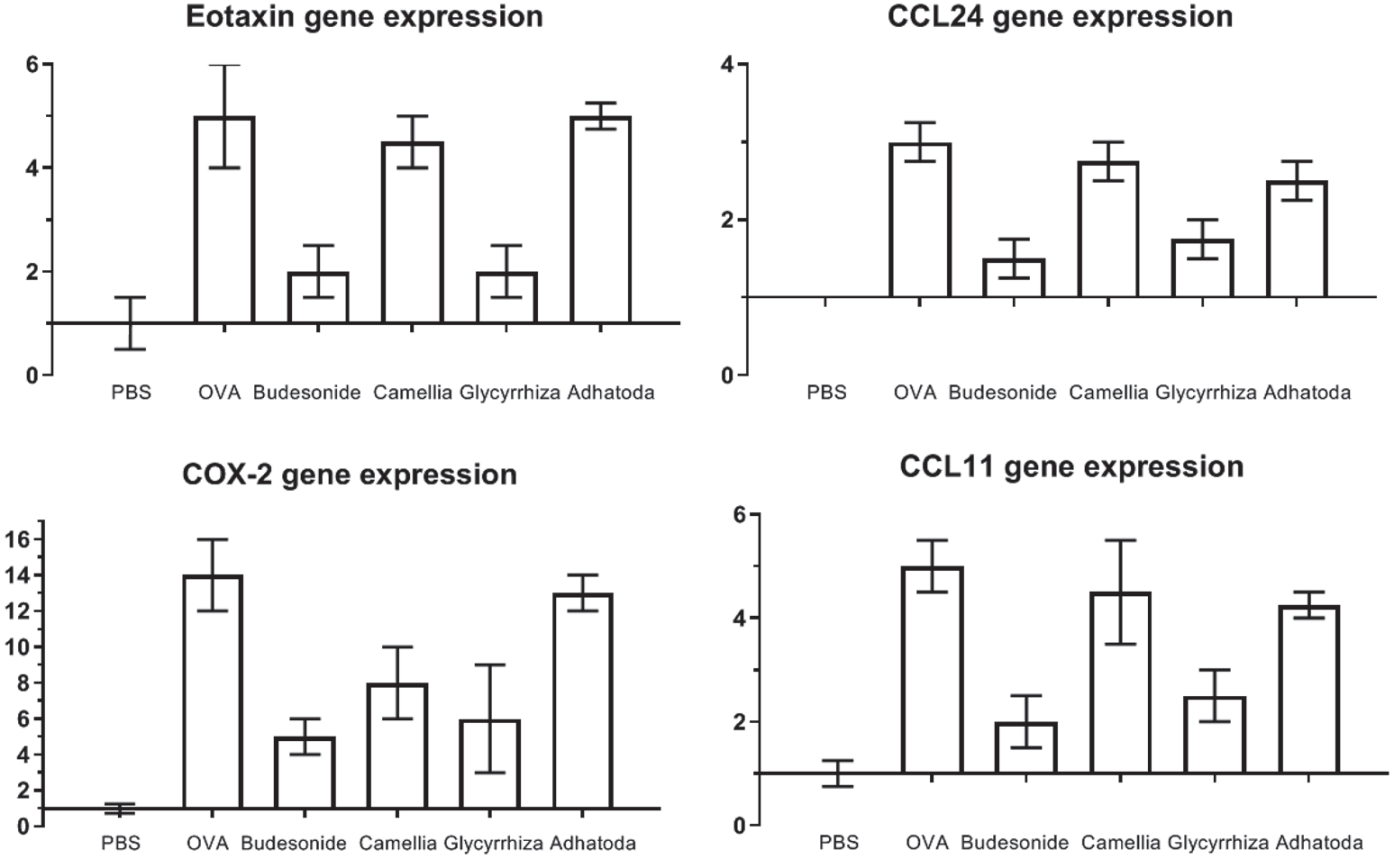
Real-time PCR. Effect of three herbal extracts on the mRNA gene expression of eotaxin, CCL11, CCL24, and COX-2 in bronchoalveolar lavage cells were determined by real-time PCR.

In the OVA group treated with Glycyrrhiza, mRNA expression of eotaxin (2 ± 0.5), CCL24 (1.75 ± 0.25), CCL11 (2.5 ± 0.5), and COX-2 (6 ± 3) were significantly decreased compared to the non-treated OVA group (Eotaxin: 5 ± 1, CCL24: 3 ± 0.25, CCL11: 5 ± 0.5, and COX-2: 14 ± 2) *(*p < 0.05). Camellia and Adhatoda extracts had no significant effect to decrease mRNA expression of eotaxin, CCL24, and CCL11 (Figure 6). Camellia decreased COX-2 mRNA expression (8 ± 2) compared the non-treated OVA group significantly *(*p < 0.05). 

### Histological study of the lung 

**Figure 7.**
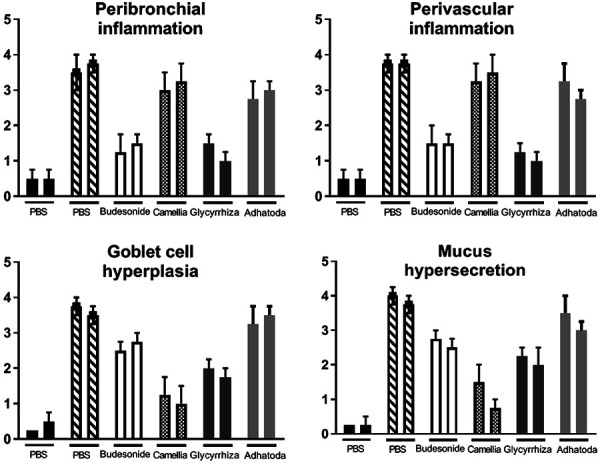
Histopathology study. Perivascular and peribronchiolar inflammation, goblet cell hyperplasia, and mucus hypersecretion were evaluated in all groups. The first column of each group is day 31 and the second is day 51.

**Figure 8.**
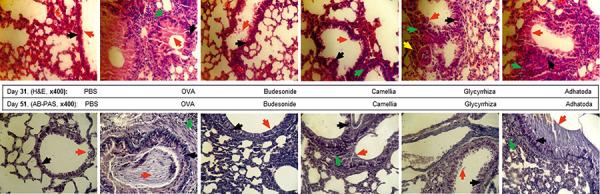
Histopathology sections. Histological sections of lung from phosphate-buffered saline, ovalbumin, budesonide, Glycyrrhiza, Adhatoda, and Camellia were produced and stained with H & E on day 31 and with AB-PAS on day 51. Eosinophil infiltration around vessels and airways (perivascular inflammation and peribronchial inflammation), hyperplasia of the goblet cells, and secretion of the mucus were analyzed. The peribronchiolar inflammation is shown by green arrows, mucus secretion by red arrows, goblet cells by black arrows, and perivascular inflammation by yellow arrows.

Histological analyses that revealed pathologic features in the Camellia- and Adhatoda-treated groups showed no significant differences in peribronchial and perivascular inflammation compared with the positive control group, but Glycyrrhiza could control and reduce eosinophil infiltration around airways and vessels on days 31 and 51 (Figure 7, 8). Mucus hyper-secretion and goblet cell hyperplasia were significantly decreased *(*p < 0.05) in Camellia- and Glycyrrhiza-treated groups compared the non-treated OVA group on days 31 and 51 (Figures 7, 8). 

## Discussion 

Allergic diseases such as allergic asthma seem to have increased in recent years. However, there are no complete cures, while in this field, herbal medicine is notable for low side effect, accessibility, and low price. Therefore, we evaluated three plants to determine whether they have an effect on allergic bio-factors. After producing an animal model of allergic asthma, the effect of these plant components were surveyed, and allergy and immunology factors were studied. We measured the levels of Th2 key cytokines and also allergy and asthma pathophysiology-related factors such as IgE.****

These three plant components are natural products with multiple biological functions, including antioxidant and anti-inflammatory effects. Previous studies have shown that Glycyrrhiza inhibits the inflammatory response, reduces the secretion of inflammatory cytokines, controls infiltration of inflammatory cells in the lung, promotes SOD expression, and enhances oxidative protection. Other species of Camellia (*C. japonica* oil) had an anti-asthmatic effect and controlled eosinophil and GATA-3 as Th2-related transcription factors, T-bet, and IL-12p40. *Adhatoda vasica Ness.* (Malabar nut) is a medicinal plant used to treat cough and asthma. Adhatoda has important effects in the treatment of respiratory disorders and may have bronchodilator compounds [11, 15, 16, 17]. In this study, we found that Adhatoda significantly attenuated the IL-4 level in BALf. In addition, Glycyrrhiza significantly attenuated the IL-5 and IL-13 levels on days 31 and 51, and Camellia significantly decreased the IL-13 level on days 31 and 51. IL-4 is related with allergic reaction, therefore, Adhatoda can be useful in the treatment and control of allergic diseases such as allergic rhinitis. Also, Adhatoda decreased histamine and IgE levels in serum. The two mentioned bio-factors are main triggers of allergic reactions, and many allergic symptoms are related with high levels of histamine and IgE. Mucus hypersecretion is forced by IL-13, and airway obstruction by mucus can lead to cough and dyspnea; therefore, Camellia can prevent mucus hypersecretion by control of IL-13 and may be useful in control of cough, especially in asthmatic patients. IL-5 leads to eosinophil maturation, activation, and migration. Control of IL-5 expression can control eosinophilic inflammation and reduce eosinophil infiltration around vessels and airways that would cure asthmatic lung. Glycyrrhiza attenuates IL-5 and reduces eosinophilic inflammation and, with decreasing of IL-13 level, leads to reduction of mucus secretion. This leads to control and treatment of asthma. The anti-allergic effect of Adhatoda is stronger than that of the two others, and the anti-inflammatory effect of Glycyrrhiza is better than that of Adhatoda and Camellia. Camellia has a better effect in control of mucus secretion. 

Previous studies showed that licochalcone A, as a component of licorice, decreases Th2 cytokine and OVA-specific IgE. However, it improves inflammation playing an anti-oxidative role in the asthmatic lung [11, 18]. In the asthmatic lung, ROS stimulate the bronchial cells to release chemokines and attract inflammatory cells to infiltrate around the bronchi. In addition, these inflammatory cells release more inflammatory cytokines and mediators, causing oxidative stress and damaging the lung. Glycyrrhiza can reduce ROS levels and expression of the chemokines CCL11 and CCL24. It can also reduce the activation of eosinophils. Furthermore, Glycyrrhiza inhibits the ROS release from inflamed bronchial cells and inflammation-related chemokine production, reducing cell damage. Glycyrrhiza increases radical-scavenging and promotes nuclear Nrf2 production and HO-1 expression. Thus, it has a protective potential against oxidative stress and can decrease hyperplasia of the goblet cell and mucus secretion and can prevent bronchial obstruction by blocking IL-13 expression in the lung. Interestingly, Glycyrrhiza reduces airway inflammation [19, 20, 21]. We found that Glycyrrhiza decreased gene expression of eotaxin, CCL24, CCL11, and COX-2. COX-2 mRNA expression was also decreased by Camellia. Therefore, Glycyrrhiza is powerful in controlling and reducing these factors, especially in allergic asthma conditions. 

A high number of eosinophils infiltrated the lungs and airways of asthmatic mice, and eosinophils release major base proteins, causing severe allergic reactions and, with releasing eosinophil cationic protein and eosinophil peroxidase, cause inflammation and damage in lung. Therefore, Glycyrrhiza has a great effect on improving the respiratory function of asthmatic patients. Our results confirm that Glycyrrhiza reduces goblet cell hyperplasia and mucus hypersecretion mainly by inhibiting IL-13 expression. It also controls peribronchial and perivascular eosinophilic inflammation by inhibiting IL-4 and IL-5 expression. IL-4 activates B cells to secrete IgE, and IgE binds to mast cells, mast cells activated by IgE can release histamine after re-expose with allergen, which causes allergic and inflammatory responses in asthma patients. Importantly, Glycyrrhiza reduced IL-4 production, improving the pathological features of asthma. Glycyrrhiza inhibits IL-5 production in the airways and reduces the inflammation via control of eosinophil migration [22]. GATA-3 plays a critical role in Th2 cell differentiation and cytokine expression. This regulates Th2 not only by direct binding to the Th2 cytokines promoter (IL-4, 5, and 13 genes) at the transcription level, but also by involvement in the remodeling of chromatin structures. The mRNA expression of GATA-3 is increased in airways of asthmatic patients, and the number of T cells in BALf expressing GATA-3 transcripts is correlated with AHR [23]. In our study, Glycyrrhiza and Camellia reduced the levels of GTP, but these reductions were not significant. Glycyrrhiza can reduce GOT levels in asthmatic mice on day 51. So, to be effective, Glycyrrhiza should be used over a longer time to control chronic factors of asthma. 

L-theanine, an isolated amino acid from green tea has anti-inflammatory and antioxidative activity. A study in 2017 showed that theanine can modulate airway inflammation and oxidative stress of asthma in a murine model, and treatment with L-theanine attenuated the extensive infiltration of inflammatory cells into bronchi. It also inhibited mucus, MCP-1, IL-IL-4, IL-5, IL-13, TNF-α, INF-γ, and IgE production, attenuated the generation of ROS, and activates NF-κB and matrix metalloprotease-9 [24]. CsMYB73, which belongs to subgroup 22 of the R2R3-MYB family is a transcriptional repressor involved in L-theanine biosynthesis [25]. Mucus hypersecretion and goblet cell hyperplasia were decreased in Camellia- and Glycyrrhiza-treated asthmatic groups, and Glycyrrhiza can control and reduce eosinophil infiltration. 

Previous studies showed that oral administration of Adhatoda stopped allergic symptoms and allergic rhinitis associated with bronchial asthma [26, 27]. The Adhatoda-treated group showed significant differences in changing of allergic factors that trigger allergic asthma attacks and the related problems, and Adhatoda could reduce the levels of these factors. The positive results from animal studies may help to design new treatments for human patients to control asthma symptoms and decrease the costs of healthcare and improve the quality of life with introducing effective treatment. Glycyrrhiza as main anti-inflammatory treatment, Adhatoda as anti-allergic, and Camellia as anti-mucus-releasing treatment can be used in allergic asthma attacks according to clinical signs. 

## Funding 

None. 

## Conflict of interest 

None. 

**Table 1.** Used primers sequences.

Gene	5`-3`	Primer
COX-2	Forward	ACCAGCAGTTCCAGTATCAGA
	Reverse	CAGGAGGATGGAGTTGTTGTAG
CCL24	Forward	AGGCAGTGAGAACCAAGT
	Reverse	GCGTCAATACCTATGTCCAA
CCL11	Forward	GGCTTCATGTAGTTCCAGAT
	Reverse	CCATTGTGTTCCTCAATAATCC
Eotaxin	Forward	CTGCTCACGGTCACTTCCTT
	Reverse	GGGGTCAGCACAGATCTCTT
